# The Second Victim Phenomenon After a Clinical Error: The Design and Evaluation of a Website to Reduce Caregivers’ Emotional Responses After a Clinical Error

**DOI:** 10.2196/jmir.7840

**Published:** 2017-06-08

**Authors:** José Joaquín Mira, Irene Carrillo, Mercedes Guilabert, Susana Lorenzo, Pastora Pérez-Pérez, Carmen Silvestre, Lena Ferrús, Spanish Second Victim Research Team

**Affiliations:** ^1^ Alicante-Sant Joan Health District, Universidad Miguel Hernández Elche (Alicante) Spain; ^2^ Universidad Miguel Hernández Elche (Alicante) Spain; ^3^ Hospital Universitario Fundación Alcorcón Madrid Spain; ^4^ Patient Safety Observatory, Andalusian Agency for Health Care Quality Sevilla Spain; ^5^ Servicio Navarro de Salud - Osasunbidea Pamplona Spain; ^6^ Consorci Sanitari Integral L’Hospitalet de Llobregat, Barcelona Spain; ^7^ Spanish Health System Spain

**Keywords:** patient safety, professionals, hospital, primary care, second victims, clinical error, e-learning

## Abstract

**Background:**

Adverse events (incidents that harm a patient) can also produce emotional hardship for the professionals involved (second victims). Although a few international pioneering programs exist that aim to facilitate the recovery of the second victim, there are no known initiatives that aim to raise awareness in the professional community about this issue and prevent the situation from worsening.

**Objective:**

The aim of this study was to design and evaluate an online program directed at frontline hospital and primary care health professionals that raises awareness and provides information about the second victim phenomenon.

**Methods:**

The design of the Mitigating Impact in Second Victims (MISE) online program was based on a literature review, and its contents were selected by a group of 15 experts on patient safety with experience in both clinical and academic settings. The website hosting MISE was subjected to an accreditation process by an external quality agency that specializes in evaluating health websites. The MISE structure and content were evaluated by 26 patient safety managers at hospitals and within primary care in addition to 266 frontline health care professionals who followed the program, taking into account its comprehension, usefulness of the information, and general adequacy. Finally, the amount of knowledge gained from the program was assessed with three objective measures (pre- and posttest design).

**Results:**

The website earned Advanced Accreditation for health websites after fulfilling required standards. The comprehension and practical value of the MISE content were positively assessed by 88% (23/26) and 92% (24/26) of patient safety managers, respectively. MISE was positively evaluated by health care professionals, who awarded it 8.8 points out of a maximum 10. Users who finished MISE improved their knowledge on patient safety terminology, prevalence and impact of adverse events and clinical errors, second victim support models, and recommended actions following a severe adverse event (*P*<.001).

**Conclusions:**

The MISE program differs from existing intervention initiatives by its preventive nature in relation to the second victim phenomenon. Its online nature makes it an easily accessible tool for the professional community. This program has shown to increase user’s knowledge on this issue and it helps them correct their approach. Furthermore, it is one of the first initiatives to attempt to bring the second victim phenomenon closer to primary care.

## Introduction

Patient safety incidents include both near misses (incidents that do not cause harm) and adverse events (incidents that do). Although the frequency of near misses in clinical practice is difficult to specify, the frequency of adverse events at hospitals in developed countries has been established at approximately 9% [1]; in developing countries, it increases to approximately 10.5% [2]. In ambulatory care, the prevalence of adverse events has been confirmed to be approximately 2% [3-4] and 5%, respectively [5]. One-half of these adverse events are usually considered to be preventable [1]. Most are related to clinical errors, which are defined as the failure of a planned action to be completed as intended or the use of a wrong plan to achieve an aim [6]. These include system failures and human errors.

Safety incidents associated with clinical errors have a negative emotional impact on patients, but also on the health professionals thought to be involved in them. The term *second victim* is used to describe the experience of the health professional who becomes emotionally overwhelmed as a result of being involved in an incident affecting patient safety [7,8]. The view of health care organizations as *third victims* was introduced because safety incidents may damage the reputation of and reduce trust in health care organizations [9].

Mitigating the impact from these incidents in patients, the health organization, and its professionals is a responsibility of managers and middle managers in the health organizations [10]. One reason is to prevent the same incident from reoccurring [11] and another is to create a proactive culture of safety that creates conditions to alleviate their impact [12].

### Impact of Incidents for the Safety of Professionals

Among second victims, fear from legal consequences deriving from the harm done to the patient, fear of damage to their professional reputation, feelings of guilt, doubts about their own abilities for making clinical decisions, anxiety, and mood swings are frequent [7,13-15]. In some cases, these situations can progress toward posttraumatic stress disorder [16].

Among professionals, suspicion—if not fear—persists in disclosing what happened to patients due to the consequences that may result from such conversations [17,18]. Most professionals do not know what to do after an adverse event occurs, nor do they feel prepared for informing the patient [19,20]. They also question the support they would receive from their institution and colleagues [21-23].

### Frequency of the Second Victim Phenomenon

Incident severity, its consequences, and individual variability influence the impact of the adverse event in professionals and make the number of second victims vary.

In the United States and Canada, it has been estimated that between 30% [24] and 43% [25] of professionals have experienced a negative emotional response following an incident. In one recent study carried out in Australia, 76% of the professionals involved in either a near miss or an adverse event were seen to be emotionally affected by the incident [26]. As for Spanish hospitals, as much as 69% of nurses and 77% of physicians had, either firsthand or through close colleagues, experienced being the second victim within the preceding five years [27]. In primary care, these figures varied between 55% for nurses and 67% for physicians [27]. In Belgium, Van Gerven et al [28] analyzed the magnitude of the impact among professionals, its evolution over time, and the factors that contribute to minimizing such impact, arriving at the conclusion that health organizations might anticipate this impact and plan for dealing with the second victim phenomenon.

### The Help Second Victims Count On

Assistance for second victims is not part of the actions planned to be carried out when an incident affecting patient safety occurs in hospitals [9,29,30], and there are no interventions designed for primary care [29]. Professionals feel unprotected by their institutions [31-33]. Only a few hospitals have developed their own intervention programs so, thus far, the extent of intervention programs in health systems is limited.

### Intervention Programs in the Literature

Two approaches in interventions have been described. On the one hand, interventions are centered on the incident [11]. On the other, they focus on dealing with the emotional consequences of the incident [24,34]. These interventions require a positive attitude, empathy toward the second victim, and awareness about the issue of clinical errors that may occur at any moment.

Scott [24], who leads the forYOU program at the University of Missouri Health System, has described the stages that a health professional goes through subsequent to an adverse event. According to this research, only 10% of second victims require specialized mental health services. Above all, most professionals in the first moments after an incident need to talk to a colleague, be relieved of care obligations for the time being, feel respect and empathy from others, and feel supported by their institution [24].

The work group of Wu [34] at Johns Hopkins Hospital has also developed an intervention program to help second victims in adverse events. Theirs is called RISE (Resilience in Stressful Events) and it is based on the fact that most professionals involved in an adverse event need to talk to a colleague, which is usually sufficient for coping with the emotional impact in most cases.

These interventions are meant to be activated after an adverse event occurs. Actions preventive in nature have not been designed for direct care professionals or for middle managers to become aware of the problem and learn how to address it.

The literature has emphasized that professionals do not know how to act after an adverse event and that most health centers do not have protocols in place or provide instructions on how to support second victims [9,27-30]. The second victim phenomenon is unknown to a large number of health professionals and managers, and interventions designed to raise awareness in professionals about the problems presented by near errors and adverse events are nonexistent. There are also not any for providing that first support that second victims need either. A need exists for intervention programs to reinforce the proactive culture of safety at health centers and to promote natural support structures among professionals that would be activated if needed after a patient safety incident.

### Study Objective

This study’s purpose was to develop and assess an online awareness and information program on the second victim phenomenon directed at health professionals in direct contact with patients at both hospitals and primary care. Such a program should provide demonstrations on how to act with colleagues and patients during the first moments after a severe incident for patient safety. This intervention was initially designed for Spanish frontline health care professionals.

### Specific Intervention Objectives

The specific objectives included:

1. Facilitate information and training for a large number of health professionals about the issue of second victims at a reduced cost.
2. Describe emotional reactions and common behavior after being involved in an adverse event and that characterize the second victim phenomenon.
3. Describe correct and incorrect actions of how to act after an adverse effect in order to respect the rights of patients and support the second victim.
4. Act in the area of primary care, expanding the extent of studies that up to now have only taken place at hospitals.

## Methods

This is a design and evaluation study of a website devised to mitigate the impact from severe adverse events in hospitals and primary care professionals. This intervention was named Mitigating Impact in Second Victims (MISE).

**Figure 1:**
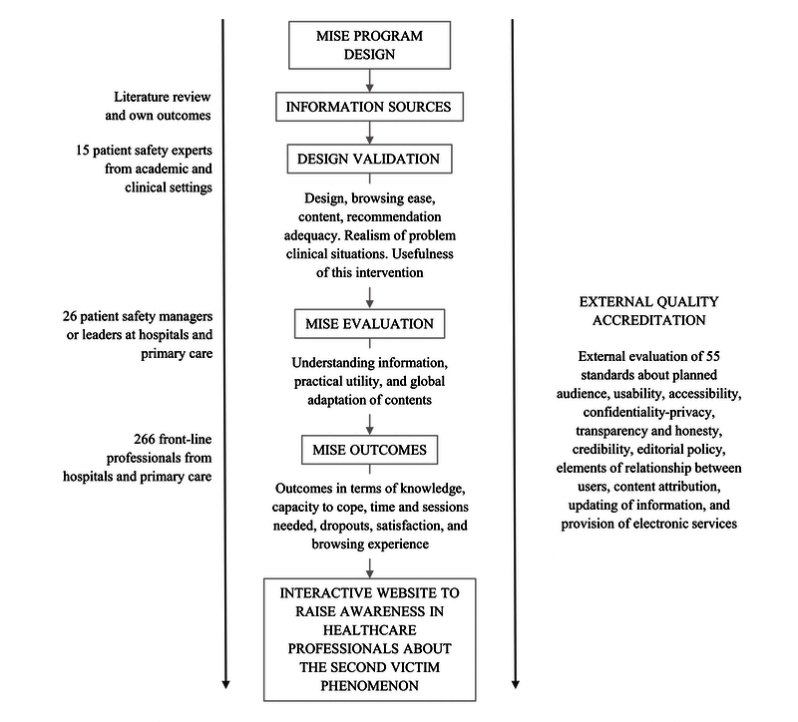
Study phases.

The phases in the design and evaluation of the website and the preventive intervention program to mitigate the initial impact from an error in health professionals (MISE) are described in Figure 1.

### Website Design and Mitigating Impact in Second Victims

A website was designed that hosted an awareness and preventive intervention program (MISE) to mitigate the impact from errors in frontline professionals [35]. The website was structured around two menus: the main menu contained general information on the second victim phenomenon regarding the different actors involved (with sections entitled “Professionals,” “Patients and Family,” “Health Managers,” “Safety Coordinators,” and “Insurers”), and a secondary menu with information related to the project and its outcomes, in addition to international studies (sections entitled “Presentation,” “Who we Are,” “Project Timetable,” “Definitions,” “News,” “Publications of Interest,” “Reviews and Comments,” and “Project Outcomes”). Access to MISE was gained by clicking on the upper right-hand corner on all website pages [36]

Based on patient safety literature, and that specifically on second victims, a preventive intervention program was designed with informative and demonstrative contents.

A review of review studies relating to open disclosure and second and third victims published in English or Spanish between 2000 and 2015 was conducted. This search was carried out using MEDLINE and Web of Knowledge. Keywords used for the review included a combination of the terms “patient safety” and “adverse event” with the following terms: emotional response, impact, professionals, second victim, third victim, and open disclosure. This yielded 22 possible documents on second victims and 83 on open disclosure. A review of health care organization websites was also conducted. This review included proposals of applied programs, checklists, and algorithms about interventions in the aftermath of an adverse event to support patient and second victims. This was carried out with the Google meta-search engine using the same descriptors. Only websites in English or Spanish were considered. A total of 16 websites were reviewed, two from Europe and the rest from the United States.

A group of 15 health professionals with at least 10 years’ experience in quality and safety participated as a promoting group, and they were responsible for identifying content and elements of relevant information and example situations to be included in MISE. Chosen first to be disseminated were elements of patient safety information, specifically on second victims. Then, an index for MISE was created. Third, problem situations were selected for the demonstrative program that were then ultimately acted out by professional actors and recorded on video.

MISE was structured in two packages, one informative and the other demonstrative. The informative package offered information on basic patient safety concepts (incidents for patient safety, incidents without harm, near errors, adverse events), along with the frequency, causality, consequences, avoidability, and other characteristics of adverse events at hospitals and within primary care. Furthermore, it introduced the concepts of second and third victims and the results from research on the impact of adverse events.

The demonstrative intervention package provided a description of the emotional consequences from adverse events in professionals (affective and emotional, in clinical decision making, loss of self-esteem and professional reputation, in relationships with other professionals, with the family, legal implications) and recommendations for action following an adverse event, specifically about how to interact with the patient and their family (open disclosure), how to support a colleague who becomes a second victim, and how to personally cope with the second victim experience. This package included 15 demonstrative videos that showed what and what not to do in different clinical situations linked to errors (Table 1).

**Table 1:** Situations represented in the videos included in the demonstrative intervention packet.

Element covered	Situation	Number of videos
Patient information; crisis communication	A group of patients is to be vaccinated without receiving warning that an electrical power outage occurred the night before, which broke the vaccination cold chain (system failure).	2
Information for the patient and the patient’s family	Surgical material is left inside a patient that requires reintervention to extract the forgotten object.	2
Support for the second victim; information for the family of a deceased patient (the person disclosing the information is a professional other than the one involved in the event)	A patient whose condition is severe dies after presenting postintubation laryngeal stenosis. The physician attending the patient exhibits emotional affection after committing an omission mistake during the patient’s resuscitation.	2
Role of peers and supervisors in supporting the second victim; notification of incidents without harm	A nurse is emotionally affected after committing a route of administration error for a medicine that did not result in serious consequences for the patient.	4
Role of managers in supporting the second victim	A physician becomes a second victim after committing a diagnosis error with serious implications for the patient’s health. After identifying the error and having a substitute professional inform the patient, the patient files a complaint.	2
Preventive measures after an error deriving from a system failure	A nurse mistakenly administers to a patient a nonindicated medication due to incorrect storage of said drug.	3

**Index of the contents and their format in the website’s informative and demonstrative packages.**

**Informative package**

**What is a patient safety incident? (text)**

What is an incident without harm—near error? (text; expert video)

What is an adverse event (AE)? (text; PowerPoint presentation with voice narration)

**Description of the research project at primary care and hospitals**

What is intended?—project objectives (text)

Why this project? (text; PowerPoint presentation with voice narration)

Who are we? (text; PowerPoint presentation with voice narration)

Sources of information on patient safety (PowerPoint presentation with voice narration)

**Second victims (text)**

What is a second victim? (text; expert video)

What do we know about second victims? (text; PowerPoint presentation with voice narration)

Outcomes of our study (text; PowerPoint presentation with voice narration)

Institutional help: BICEPS, forYOU, Johns Hopkins Hospital protocol (text; PowerPoint presentation with voice narration)

Spanish Ministry of Health Strategy on Patient Safety (text; link to PDF document on strategy)

What must not be done? (text)

**Third victims**

What is a third victim? (text; PowerPoint presentation with voice narration)

Outcomes of our study (text; PowerPoing video with voice turned off; link to PDF of questionnaires used in the study)

Crisis communication (PowerPoint presentation with voice narration)

What must not be done? (text)

**Demonstrative package**

Emotional, family, and work consequences in professionals from adverse events (text; PowerPoint presentation with voice narration)

**Recommendations about how to act after an adverse event (link to document guide available in both Spanish and English [37] created by the Second and Third Victims Research Group based on reviews of international papers and adaptation to particularities of the Spanish context)**

Role of managers (link to Safety Agenda Mobile app [12]; demonstrative videos on crisis communication)

Care for the first victim (text)

**Informing the patient (PowerPoint presentation)**

Open disclosure step-by-step guide (PowerPoint presentation with voice narration; demonstrative videos on informing the patient who has suffered an adverse event and the patient’s family)

**Support for the second victim (text and images)**

Role of second victim’s peers (PowerPoint presentation with voice narration)

Guide on how and how not to act (PowerPoint presentation with voice narration; demonstrative videos on the role of peers, supervisors, and managers in support of the second victim)

**How to be prepared and know what happened (text; demonstrative videos on preventive measures after error deriving from system failure)**

Root cause analysis (PowerPoint presentation with voice narration)

2.5.2. London protocol (PowerPoint presentation with voice narration)

In order to make MISE contents more dynamic, different formats were used to convey information: text, images, Portable Document Formats (PDFs), PowerPoint presentations with voice narration, videos in which a patient safety expert appears and explains a concept, demonstrative videos (simulations of situations with actors), in addition to a mobile app.

Textbox 1 lists the contents selected by the group of experts for each MISE package and the chosen format in each case for presenting such information.

First, an independent agency specializing in the evaluation of health websites completed the accreditation of its overall design, structure, organizational, and functional quality according to a certification standard [38]. This external evaluation was led by technical personnel from that agency with experienced auditors.

Second, MISE was evaluated by academic and professional safety experts who themselves were responsible for the services of patient quality and safety at hospitals and within primary care in Spain.

Then, MISE was evaluated by a group of professionals who voluntarily followed this program between November 2015 and February 2017. Moreover, they considered the usefulness of MISE for improving information about the second victim phenomenon and what to do after an adverse event. For this purpose, the participants answered a series of knowledge tests.

### Website Certification (External Assessment)

The research team assessed the website (self-assessment), following the quality standards of the Andalusian Agency for Healthcare Quality [38]. It was then evaluated externally following the accreditation program for health-related websites of this agency.

This accreditation procedure consisted of 55 standards (31 were required, 10 were recommended, but in order to receive Advanced Accreditation, 8 of these must be met; the remaining 14 are voluntary commitments) that address the following aspects: usability, accessibility, confidentiality-privacy, transparency, credibility, editorial policy, elements related to the Web user, attribution of contents, updating of information, and provision of electronic services.

This evaluation was based on a double procedure of self-assessment and external evaluation. The self-assessment permitted interactive identification of elements from the webpage in need of improvements. By following this system, changes in the design and browsing conveniences were introduced into the website. The subsequent external evaluation ensured compliance with the criteria based on webpage operation, content, and resources.

### Suitability of Mitigating Impact in Second Victims by Patient Safety Experts

A group of 26 health professionals who were managers of patient safety services assessed MISE. This professional profile was chosen because their criteria were thought to be the best for assessing the program’s focus and content. To complete their assessment, they were allowed to freely explore the program for several weeks. This group included physicians and nurses from the health services at hospitals and within primary care, and the participants had more than three years’ professional experience in patient safety. They completed an online questionnaire after being called on the telephone to request their participation and to provide an email address in order to send them an online questionnaire link. Their responses were anonymous and voluntary, and these experts assessed the comprehension of the information, practical usefulness of the contents, and overall suitability on a scale from 0 to 10, with 10 representing the highest possible assessment.

### Evaluation of Mitigating Impact in Second Victims

To assess the acquisition of knowledge by intervention program users, three objective tests with pre-established response options at different points in the program were included. Specifically, two tests with pre- and posttest measures were prepared, and these included a total of 20 questions. The first evaluated the additional knowledge gained after completing the informative package (12 items); the second evaluated additional knowledge gained in the demonstrative package (8 items). These test questions consisted of statements with true/false answers. A third series of questions was used, prepared from the demonstrative videos, in which the user had to choose the correct action between two response options. These additional questions also permitted assessing the program’s effectiveness in terms of the ability to discriminate between how to act in each situation. These consisted of a total of 25 questions and were answered only after the videos were watched.

Once they finished the program, the participants assessed MISE in terms of comprehending the information, the practical value of its contents, and its overall suitability. Furthermore, the following measures were also considered: the number of connections required to finish the program, total time invested to finish it, average time of each connection, number of program dropouts, and correct answers on the knowledge test (pre- and postmeasures and questions on the situations represented in the demonstrative videos).

### Participants in Mitigating Impact in Second Victims Evaluation

Safety professionals from nine autonomous health services in Spain participated in this study. As a country, Spain has 17 autonomous communities, and each has its own health system. The nine participating health services account for 75% of all care activity occurring at hospitals and within primary care in the country.

A sample of 351 professionals from hospitals and primary care within these health services were asked to voluntarily participate. A minimum sampling size of 245 participants was determined, considering a sampling error of 5%, 80% correct answers on the questions, and 70% participation for a 95% confidence level. Quality and safety managers at the centers collaborated in recruiting participants by extending invitations to their hospital and health center staff to participate. To enter the system, the participants had to use a personal password to identify themselves; this way, they could continue participating in the MISE program as time permitted.

Before entering the system, the participants were informed about the study’s scope, objectives, and method, in addition to the conditions for their participation. They granted their consent as a requirement for access.

### Simple Blind System

Two databases were employed. The first contained the keys used by each participant, separate from the remaining databases. The second contained the anonymized registries of the participants’ responses. Only the authorized webmaster had access to the participant databases and no personal data in the response database were linked to the pre-post measures.

### Statistics

A student *t* test with repeated measures (intrasubject comparisons) was used to assess the intervention’s effectiveness by comparing the pre-post measures. A McNemar test was used to assess the impact of the videos.

### Investigation Ethics

This study was approved by the Ethics Committee of the San Juan de Alicante Hospital (Alicante, Spain).

## Results

### Accreditation

The external evaluation recognized the entire research project website (including MISE) as a health website and awarded it the level of Advanced Accreditation on November 25, 2016. To gain this recognition, 100% of the required standards were satisfied (31/31) along with 80% or more of those recommended (8/10), surpassing the thresholds required by the evaluated standards. Overall, the website complied with 73% (40/55) of those standards. Four standards were not applicable because they referred to patients’ rights and the treatment of their health information (the website is directed solely at health professionals and does not allow compiling clinical data of patients), advertising content (absent on the website), virtual health communities (interaction between users is not included among the website’s objectives), and the provision of electronic services (the website is not used as a tool for carrying out commercial activities). If these four standards are discounted, the percentage of compliance with the requirements increases from 73% to 78%.

Its strengths were related to identifying the recipients, usability, confidentiality-privacy, transparency and honesty, credibility, attribution of contents, and updating of information. Areas for improvement were related to elements related to website users, accessibility, editorial policy, and usability (Table 2).

**Table 2:** Results of the website’s external accreditation.

Element evaluated (grouping of standards)	Standards, n	Compliance, n (%)
Target audience	1	1 (100)
Usability	11	8 (73)
Accessibility	20	13 (65)
Confidentiality-privacy (privacy and data protection)	4	4 (100)
Transparency and honesty	3	3 (100)
Credibility	2	2 (100)
Editorial policy	6	4 (67)
Elements related to website users	3	0 (0)
Attribution of contents	4	4 (100)
Updating of information	1	1 (100)
Total	55	40 (73)

**Table 3:** Description of the user sample (N=266).

Demographics			n (%)
**Sex**			
	Male		83 (31.2)
	Female		183 (68.8)
**Professional profile**			
	Physicians		114 (42.9)
	Nurses		120 (45.1)
	Other health care professionals		32 (12.0)
**Medical department**			
	**Hospital**		211 (79.3)
		Physicians from hospitals	174 (82.5)
		Surgeons from hospitals	37 (17.5)
	Primary care		55 (20.7)
**Experience**			
	<1 year		30 (11.3)
	B1 and 3 years		11 (4.1)
	>3 years		225 (84.6)

**Table 4:** MISE evaluation by participating professionals (N=266).

Element		Datum
MISE all pages visited, n (%)		263 (98.9)
MISE dropouts, n (%)		12 (4.5)
Days to complete, mean (SD)		72.8 (40.3)
Number of MISE connections to complete program, mean (SD)		11.4 (8.3)
Connection time per session (minutes), mean (SD)		25 (17)
**MISE assessment (scale from 0 to 10), mean (SD)**		
	Comprehension of the information	8.9 (1.1)
	Practical value of the contents	8.8 (1.2)
	General assessment	8.8 (1.3)

### Evaluation by National Patient Safety Experts

Twenty-six patient safety experts from four autonomous health services assessed MISE (100% response rate). Of these, 92% (24/26) positively assessed the ease of browsing and following the programmed activities, 88% (23/26) positively assessed the comprehension of the contents (mean 8.8, SD 0.9), and 92% (24/26) did likewise for the practical value of the designed intervention (mean 8.7, SD 1.1).

### Participation and Evaluation of the Activity

In all, 266 of 351 professionals (75.8% response rate) followed the activities proposed in MISE; of them, 183 were women and 83 were men (Table 3).

Those who completed MISE viewed 99% of its pages (Table 4). On average, two months were required to finish reading its content, watching its videos, and completing the activities. The mean number of connections needed to complete MISE was 11.4 (SD 8.3), and these ranged between 1 and 57. The mean length of each connection was almost 30 minutes. MISE dropouts (those who quit without viewing at least 70% of its pages) were less than 5% (12/266, 4.5%).

Mitigating Impact in Second Victims was highly rated by the professional users, and they awarded it almost nine points out of a maximum of 10 (Table 4). Only two of 266 participants (0.7%) awarded it less than six points for comprehension and usefulness of the program’s information.

### Postmeasures on the Program’s Effectiveness: Pre-Post Comparisons

Participants who completed MISE increased their level of knowledge on patient safety terminology (near misses, adverse events, and sentinel events), prevalence and impact of adverse events and errors (first, second, and third victims), support models for the second victim, and the recommended actions following a severe adverse event. There was a significant difference in the pre- and postmeasures of the knowledge test of information about basic patient safety concepts, prevalence and nature of adverse events, and second victims (informative package). Out of a maximum of 12, the premeasure mean was 6.9 (SD 2.0) and the postmeasure mean was 8.8 (SD 1.6; *t*_265_=–10.0, *P*<.001). There was also a significant difference in the pre- and postmeasures of the knowledge test of what to do after an adverse event or error (demonstrative package). Out of a maximum of 8, the premeasure mean was 6.3 (SD 1.5) and the postmeasure mean was 7.2 (SD 1.0; *t*_265_=–6.2, *P*<.001).

The correct answers on the knowledge tests did not vary between physicians and nurses in all cases (general knowledge test: *P*=.27; informative test package, MISE: *P*=.13; and demonstrative test package, MISE: *P*=.89).

After watching the problem situations (demonstrative videos), most test questions were answered correctly with the exception of situations representing a system failure. For these, 13.9% (37/266) of the users attributed the event of the hypothetical situation shown in the video to a human error instead of a system failure, and they considered that such failures can always be prevented (Table 5).

### Knowledge Test Error Analysis

In the pretest, questions in which the answer given was incorrect more than 50% of the time had to do with the number of professionals involved in this type of event, patient safety concepts (definitions of incidents without harm and the second victim), the preventive ability against system failures, and procedures for crisis communication and open disclosure (who and how). In all these cases, the participants answered these questions as being true when in fact the correct answers were false (Table 6).

**Table 5:** Number of correct answers after watching demonstrative videos on what and what not to do (N=266; total questions answered=25).

Video content	Possible correct answers, n	Mean (SD)	Participants with all correct answers, n (%)
Forgotten gauze video	3	2.8 (0.4)	211 (79.3)
Extubation error video	4	3.9 (0.5)	244 (91.7)
Crisis communication video	3	3.0 (0.3)	257 (96.6)
Video on support for second victim by peers	6	5.9 (0.5)	234 (88.0)
Video on support for second victim by managers	3	2.8 (0.4)	219 (82.3)
System failure video	2	1.2 (0.7)	97 (36.5)
Human error video	2	1.7 (0.4)	198 (74.4)

**Table 6:** Analysis and evolution of the errors (&gt;50%) in the knowledge tests.

Item		Error, %		Difference	*P*^a^
		Pretest	Posttest		
**Informative package**					
	If a patient is prescribed a medication that his/her medical record says he/she is allergic to, but on that occasion no harm results, we are talking about a near incident	63.3	52.1	–11.2	.03
	According to available data, close to 40% of health professionals are seen as being directly involved in an adverse event every year in our country (Spain)	85.3	70.2	–15.1	<.001
	Every health professional who is seen to be directly involved in an adverse event is considered a second victim	90.9	83.5	–7.4	.21
	In crisis communication, not disclosing any information during the first 24 hours, until an in-depth analysis of what occurred is completed and detailed information becomes available, is fundamental	57.1	38.0	–19.1	.003
**Demonstrative package**					
	The most appropriate professional for informing the patient who suffered an adverse event is the person seen as being most directly involved because it is this person who knows best what happened	51.4	43.0	–8.4	.30

^a^ McNemar test.

## Discussion

### Principal Results

Mitigating Impact in Second Victims includes a set of contents that has been considered appropriate by patient safety experts. It has also shown to contribute to improving knowledge among health professionals about the second victim phenomenon. The methodology employed for disseminating this knowledge and explaining what and what not to do has been considered appropriate by the MISE participants.

Data from the MISE evaluation confirm that the program increases knowledge about the issue of second victims and how to act with a colleague when either an adverse event or near miss occurs. It also shows how to interact with patients who are victims of an adverse event, providing information on how to act and how to disclose what has occurred.

Five hours was the mean total time dedicated to complete MISE, distributed generally over 12 sessions lasting approximately 30 minutes each. This time demand is reasonable for this group of professionals and is compatible with other care responsibilities and tasks and their personal lives.

### Comparison With Previous Studies

Emotional needs immediately following incidents have been analyzed in several studies. The emotional isolation professionals find themselves in, along with the difficulty of talking about what has happened with their colleagues and the lack of protection they feel from their superiors, have been identified as two important gaps that contribute to progressing along the scale of the second victim syndrome [39]. Both aspects have been corroborated by research carried out among participants in benchmark intervention programs (forYOU or RISE) with second victims and that, in turn, pointed out that most of what second victims were searching for and would have liked to receive was support from their colleagues and the management at their centers [24,34].

We know that colleagues of second victims can do much more than what they currently do to prevent the emotional impact from safety incidents for the patient from progressing until manifesting itself as posttraumatic stress [8,23,40,41]. We also know that most professionals do not require specialized intervention to alleviate their initial emotional symptoms because sensing empathy by their colleagues can be sufficient [34]. The MISE intervention program considers these aspects and seeks to act at the base of Scott’s pyramid, where 60% of the professionals who suffer from the impact of incidents for safety as second victims are found [24].

The role of managers is crucial in two senses due to their role as a barrier and their role as a facilitator [10,28,42]. Managers should prepare the organization so that if a severe incident does occur, it is prepared to act, and this includes analysis of what happened and the recovery by and support for the patient and for the second victim as well. Furthermore, they should create a just culture [43] that permits analyzing the incident without prejudging the second victim’s role in it. Likewise, managers should facilitate organizational learning from incidents using formal and informal processes as well as reactive and proactive approaches. Incident reporting is a crucial step for improving patient safety, but frontline professionals identify barriers (ie, lack of training, undesirable repercussions, lack of feedback) that lead to underuse of incident reporting systems [44]. However, Sujan [44] found that professionals use informal processes, such as regular staff meetings, discussions with line managers, and discussions with peers, that facilitate sharing concerns and experiences that can actively contribute to improving patient safety. Organization leadership should be aware of these alternative ways of learning and promote them.

Subsequent to a severe adverse event, the second victim may become helpless and not inform the patient about the incident [45-49]. However, such attention and information on the part of the professionals, when presented in an appropriate manner, usually facilitates resolution of the crisis and prevents litigation [50]. The contents and approach of MISE aim in this direction.

### Relevance of This Study

Mitigating Impact in Second Victims is easily accessible to a large number of professionals. It is a low-cost program that can be accessed from work or home with ease.

This program responds to three deficiencies identified in the literature and in daily practice at health centers: (1) the lack of programs raising awareness and providing information about the second victim phenomenon that reaches high numbers of professionals, (2) the issue of second victims in primary care, and (3) the inexistence of structured interventions at most health centers to support professionals and patients following adverse events.

The MISE program provides information to professionals about the second victim phenomenon in nine weeks of online training in which are presented general issues and problem situations based on experiences after committing a clinical error.

The need for second victims to change their care functions could be reduced and less absenteeism linked to this phenomenon [27] could result if these professionals gain greater information and a change in attitude toward second victims. This sought-after attitudinal change would also facilitate a distinct attitude when interacting with patients in order to overcome the traditional difficulties resulting after an adverse event [51].

### Limitations

It is possible that those who followed the MISE program were professionals who are more sensitive to the issue of what severe adverse events mean for professionals. We did not possess information about the type of professionals who declined invitations to follow MISE.

A minimum sampling size was defined considering a worst case of 80% correct answers to the questions. The correct answers related to system failure did not match this assumption.

The measure of the resulting impact was based on correct answers to knowledge tests and to self-test questions after watching a set of problem situations on video. Actual adverse events that occur may involve circumstances that are different from what these videos represented.

This study was not designed to assess its effect on secondary prevention of posttraumatic stress; that is something that future research should evaluate. Thus, the effectiveness of this website in contributing to any kind of change was not assessed, and this will be done in the future. In this sense, realistic evaluation, a form of theory-driven evaluation developed by Pawson and Tilley in 1997 [52], may be a good methodology for testing MISE. Realistic explanation refutes the idea that a program works or does not work in an absolute manner and proposes that it is necessary to identify the mechanism (ie, the process of how subjects interpret and act on the intervention) by which the program works for whom and under what particular circumstances [52]. In this way, a program can be effective for achieving some outcomes or changes but not others, always depending on the context.

### Recommendations for Practice and Research

The MISE program is designed to assist intervention programs to mitigate the impact of adverse events in professionals. It is not an emotional recovery program for second victims; instead, it responds to the need for the group of professionals to understand what is felt subsequent to an adverse event. MISE also contributes to frontline professionals gaining greater awareness about the emotional needs that are experienced when an error occurs and the importance of speaking about the incident with their colleagues. It also provides ideas about how to act with the patient victim of the adverse event [37]. This way, when an adverse event does occur, MISE contributes by helping the professional affected (second victim) to face the facts and recover his/her clinical capacity and emotional balance early on.

These types of programs, along with other recommendations about what to do after an adverse event occurs, contribute to safer environments at hospitals and primary care.

Future research could analyze whether MISE modifies the frequency, which up until now is relatively low, of patients who are victims of adverse events being informed about the incident. This research could also examine the impact of MISE in the initial hours after an incident. One example of this would be whether the colleagues of the second victims gain a greater ability to listen and act appropriately to prevent the emotional escalation that may result.

## Abbreviations

MISE: Mitigating Impact in Second Victims
PDF: Portable Document Formats
RISE: Resilience in Stressful Events
